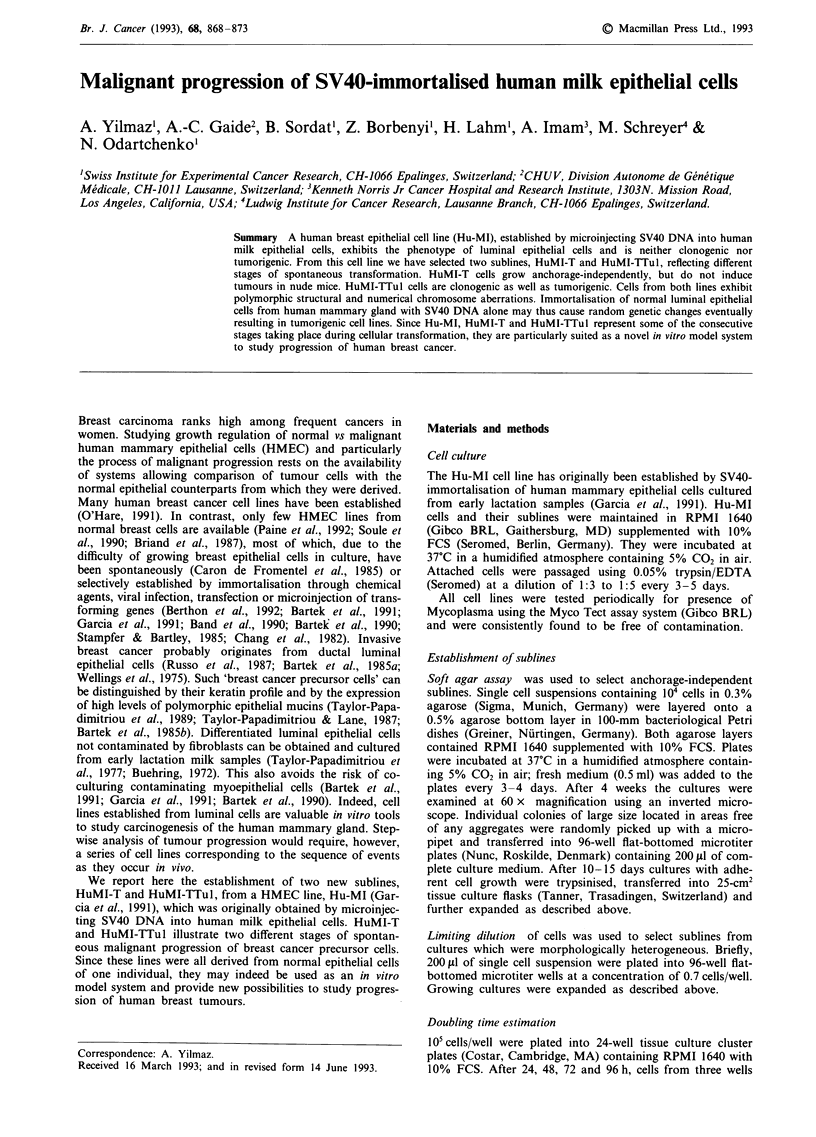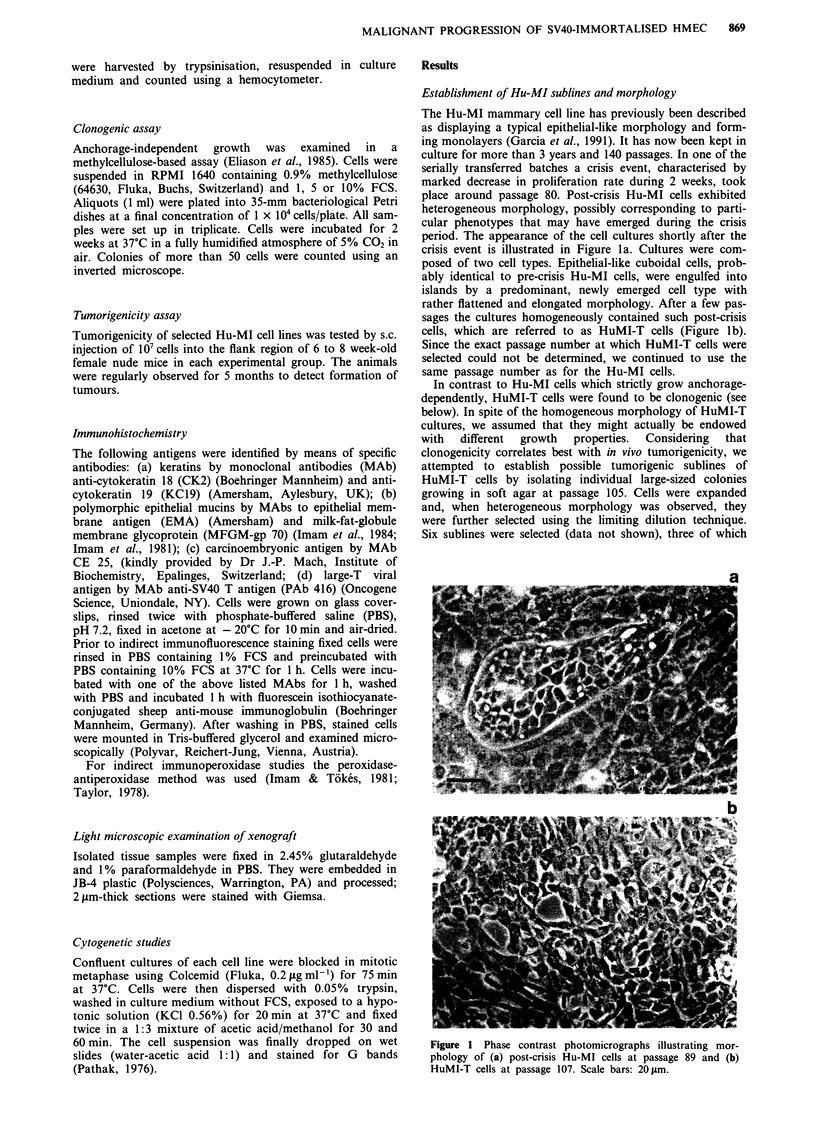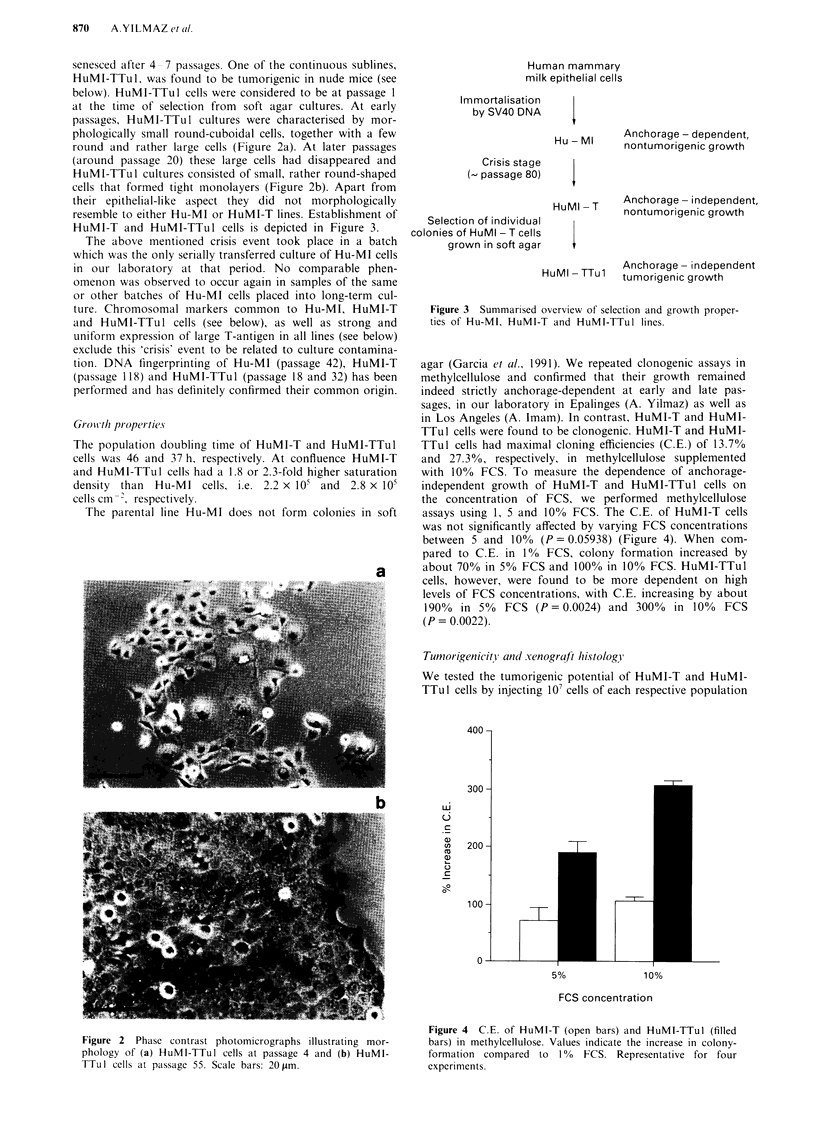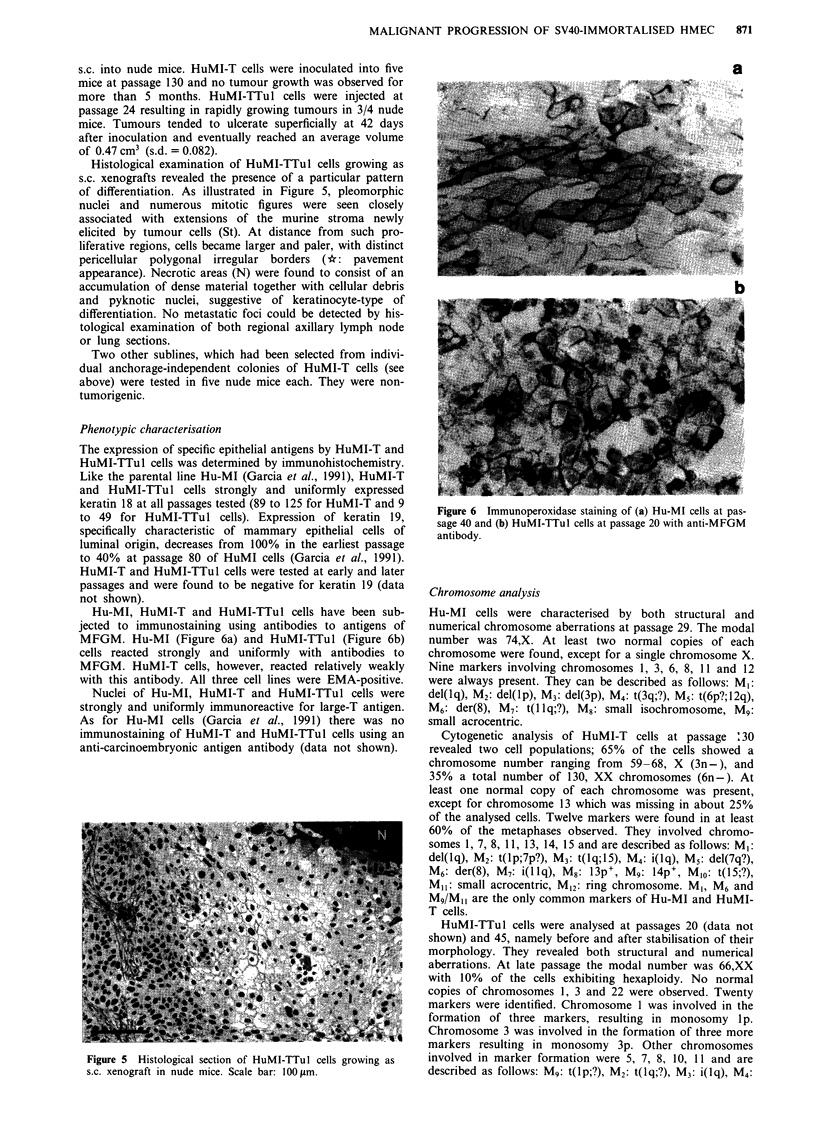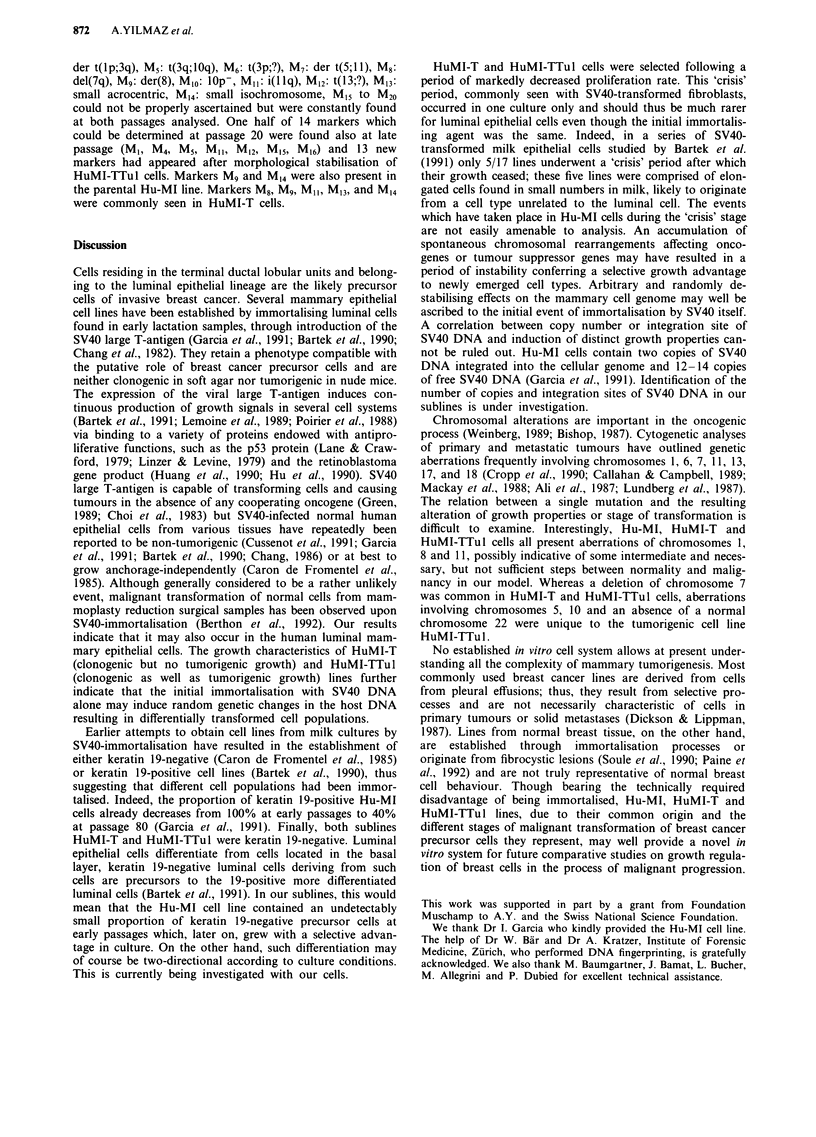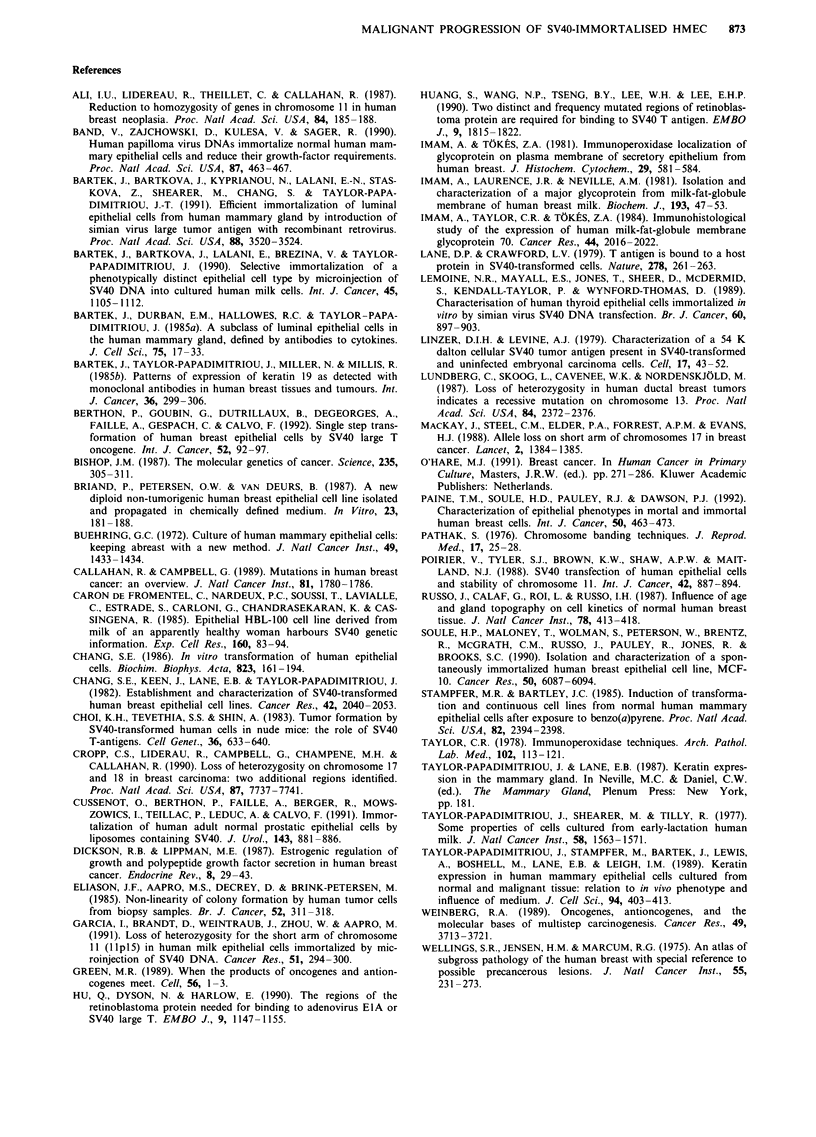# Malignant progression of SV40-immortalised human milk epithelial cells.

**DOI:** 10.1038/bjc.1993.447

**Published:** 1993-11

**Authors:** A. Yilmaz, A. C. Gaide, B. Sordat, Z. Borbenyi, H. Lahm, A. Imam, M. Schreyer, N. Odartchenko

**Affiliations:** Swiss Institute for Experimental Cancer Research, Epalinges.

## Abstract

**Images:**


Br. J. Cancer (1993), 68, 868–873 © Macmillan Press Ltd., 1993

### Malignant progression of SV40-immortalised human milk epithelial cells

A. Yilmaz[1], A.-C. Gaide[2], B. Sordat[1], Z. Borbenyi[1], H. Lahm[1], A. Imam[3], M. Schreyer[4] & N. Odartchenko[1]

[1]*Swiss Institute for Experimental Cancer Research, CH-1066 Epalinges, Switzerland;* [2]*CHUV, Division Autonome de Génétique Médicale, CH-1011 Lausanne, Switzerland;* [3]*Kenneth Norris Jr Cancer Hospital and Research Institute, 1303N. Mission Road, Los Angeles, California, USA;* [4]*Ludwig Institute for Cancer Research, Lausanne Branch, CH-1066 Epalinges, Switzerland.*

**Summary** A human breast epithelial cell line (Hu-MI), established by microinjecting SV40 DNA into human milk epithelial cells, exhibits the phenotype of luminal epithelial cells and is neither clonogenic nor tumorigenic. From this cell line we have selected two sublines, HuMI-T and HuMI-TTu1, reflecting different stages of spontaneous transformation. HuMI-T cells grow anchorage-independently, but do not induce tumours in nude mice. HuMI-TTu1 cells are clonogenic as well as tumorigenic. Cells from both lines exhibit polymorphic structural and numerical chromosome aberrations. Immortalisation of normal luminal epithelial cells from human mammary gland with SV40 DNA alone may thus cause random genetic changes eventually resulting in tumorigenic cell lines. Since Hu-MI, HuMI-T and HuMI-TTu1 represent some of the consecutive stages taking place during cellular transformation, they are particularly suited as a novel *in vitro* model system to study progression of human breast cancer.

Breast carcinoma ranks high among frequent cancers in women. Studying growth regulation of normal *vs* malignant human mammary epithelial cells (HMEC) and particularly the process of malignant progression rests on the availability of systems allowing comparison of tumour cells with the normal epithelial counterparts from which they were derived. Many human breast cancer cell lines have been established (O'Hare, 1991). In contrast, only few HMEC lines from normal breast cells are available (Paine *et al.*, 1992; Soule *et al.*, 1990; Briand *et al.*, 1987), most of which, due to the difficulty of growing breast epithelial cells in culture, have been spontaneously (Caron de Fromentel *et al.*, 1985) or selectively established by immortalisation through chemical agents, viral infection, transfection or microinjection of transforming genes (Berthon *et al.*, 1992; Bartek *et al.*, 1991; Garcia *et al.*, 1991; Band *et al.*, 1990; Bartek *et al.*, 1990; Stampfer & Bartley, 1985; Chang *et al.*, 1982). Invasive breast cancer probably originates from ductal luminal epithelial cells (Russo *et al.*, 1987; Bartek *et al.*, 1985*a*; Wellings *et al.*, 1975). Such 'breast cancer precursor cells' can be distinguished by their keratin profile and by the expression of high levels of polymorphic epithelial mucins (Taylor-Papadimitriou *et al.*, 1989; Taylor-Papadimitriou & Lane, 1987; Bartek *et al.*, 1985*b*). Differentiated luminal epithelial cells not contaminated by fibroblasts can be obtained and cultured from early lactation milk samples (Taylor-Papadimitriou *et al.*, 1977; Buehring, 1972). This also avoids the risk of co-culturing contaminating myoepithelial cells (Bartek *et al.*, 1991; Garcia *et al.*, 1991; Bartek *et al.*, 1990). Indeed, cell lines established from luminal cells are valuable *in vitro* tools to study carcinogenesis of the human mammary gland. Step-wise analysis of tumour progression would require, however, a series of cell lines corresponding to the sequence of events as they occur *in vivo*.

We report here the establishment of two new sublines, HuMI-T and HuMI-TTu1, from a HMEC line, Hu-MI (Garcia *et al.*, 1991), which was originally obtained by microinjecting SV40 DNA into human milk epithelial cells. HuMI-T and HuMI-TTu1 illustrate two different stages of spontaneous malignant progression of breast cancer precursor cells. Since these lines were all derived from normal epithelial cells of one individual, they may indeed be used as an *in vitro* model system and provide new possibilities to study progression of human breast tumours.

Correspondence: A. Yilmaz.
Received 16 March 1993; and in revised form 14 June 1993.

### Materials and methods

### Cell culture

The Hu-MI cell line has originally been established by SV40-immortalisation of human mammary epithelial cells cultured from early lactation samples (Garcia *et al.*, 1991). Hu-MI cells and their sublines were maintained in RPMI 1640 (Gibco BRL, Gaithersburg, MD) supplemented with 10% FCS (Seromed, Berlin, Germany). They were incubated at 37°C in a humidified atmosphere containing 5% $CO_2$ in air. Attached cells were passaged using 0.05% trypsin/EDTA (Seromed) at a dilution of 1:3 to 1:5 every 3–5 days.

All cell lines were tested periodically for presence of Mycoplasma using the Myco Tect assay system (Gibco BRL) and were consistently found to be free of contamination.

### Establishment of sublines

*Soft agar assay* was used to select anchorage-independent sublines. Single cell suspensions containing $10^4$ cells in 0.3% agarose (Sigma, Munich, Germany) were layered onto a 0.5% agarose bottom layer in 100-mm bacteriological Petri dishes (Greiner, Nürtingen, Germany). Both agarose layers contained RPMI 1640 supplemented with 10% FCS. Plates were incubated at 37°C in a humidified atmosphere containing 5% $CO_2$ in air; fresh medium (0.5 ml) was added to the plates every 3–4 days. After 4 weeks the cultures were examined at 60 × magnification using an inverted microscope. Individual colonies of large size located in areas free of any aggregates were randomly picked up with a micropipet and transferred into 96-well flat-bottomed microtiter plates (Nunc, Roskilde, Denmark) containing 200 µl of complete culture medium. After 10–15 days cultures with adherent cell growth were trypsinised, transferred into 25-cm² tissue culture flasks (Tanner, Trasadingen, Switzerland) and further expanded as described above.

*Limiting dilution* of cells was used to select sublines from cultures which were morphologically heterogeneous. Briefly, 200 µl of single cell suspension were plated into 96-well flat-bottomed microtiter wells at a concentration of 0.7 cells/well. Growing cultures were expanded as described above.

### Doubling time estimation

$10^5$ cells/well were plated into 24-well tissue culture cluster plates (Costar, Cambridge, MA) containing RPMI 1640 with 10% FCS. After 24, 48, 72 and 96 h, cells from three wells were harvested by trypsinisation, resuspended in culture medium and counted using a hemocytometer.

*Clonogenic assay*

Anchorage-independent growth was examined in a methylcellulose-based assay (Eliason *et al.*, 1985). Cells were suspended in RPMI 1640 containing 0.9% methylcellulose (64630, Fluka, Buchs, Switzerland) and 1, 5 or 10% FCS. Aliquots (1 ml) were plated into 35-mm bacteriological Petri dishes at a final concentration of $1 \times 10^4$ cells/plate. All samples were set up in triplicate. Cells were incubated for 2 weeks at 37°C in a fully humidified atmosphere of 5% $CO_2$ in air. Colonies of more than 50 cells were counted using an inverted microscope.

*Tumorigenicity assay*

Tumorigenicity of selected Hu-MI cell lines was tested by s.c. injection of $10^7$ cells into the flank region of 6 to 8 week-old female nude mice in each experimental group. The animals were regularly observed for 5 months to detect formation of tumours.

*Immunohistochemistry*

The following antigens were identified by means of specific antibodies: (a) keratins by monoclonal antibodies (MAb) anti-cytokeratin 18 (CK2) (Boehringer Mannheim) and anti-cytokeratin 19 (KC19) (Amersham, Aylesbury, UK); (b) polymorphic epithelial mucins by MAbs to epithelial membrane antigen (EMA) (Amersham) and milk-fat-globule membrane glycoprotein (MFGM-gp 70) (Imam *et al.*, 1984; Imam *et al.*, 1981); (c) carcinoembryonic antigen by MAb CE 25, (kindly provided by Dr J.-P. Mach, Institute of Biochemistry, Epalinges, Switzerland; (d) large-T viral antigen by MAb anti-SV40 T antigen (PAb 416) (Oncogene Science, Uniondale, NY). Cells were grown on glass coverslips, rinsed twice with phosphate-buffered saline (PBS), pH 7.2, fixed in acetone at − 20°C for 10 min and air-dried. Prior to indirect immunofluorescence staining fixed cells were rinsed in PBS containing 1% FCS and preincubated with PBS containing 10% FCS at 37°C for 1 h. Cells were incubated with one of the above listed MAbs for 1 h, washed with PBS and incubated 1 h with fluorescein isothiocyanate-conjugated sheep anti-mouse immunoglobulin (Boehringer Mannheim, Germany). After washing in PBS, stained cells were mounted in Tris-buffered glycerol and examined microscopically (Polyvar, Reichert-Jung, Vienna, Austria).

For indirect immunoperoxidase studies the peroxidase-antiperoxidase method was used (Imam & Tökés, 1981; Taylor, 1978).

*Light microscopic examination of xenograft*

Isolated tissue samples were fixed in 2.45% glutaraldehyde and 1% paraformaldehyde in PBS. They were embedded in JB-4 plastic (Polysciences, Warrington, PA) and processed; 2 μm-thick sections were stained with Giemsa.

*Cytogenetic studies*

Confluent cultures of each cell line were blocked in mitotic metaphase using Colcemid (Fluka, 0.2 μg $ml^{-1}$) for 75 min at 37°C. Cells were then dispersed with 0.05% trypsin, washed in culture medium without FCS, exposed to a hypotonic solution (KCl 0.56%) for 20 min at 37°C and fixed twice in a 1:3 mixture of acetic acid/methanol for 30 and 60 min. The cell suspension was finally dropped on wet slides (water-acetic acid 1:1) and stained for G bands (Pathak, 1976).

### Results

*Establishment of Hu-MI sublines and morphology*

The Hu-MI mammary cell line has previously been described as displaying a typical epithelial-like morphology and forming monolayers (Garcia *et al.*, 1991). It has now been kept in culture for more than 3 years and 140 passages. In one of the serially transferred batches a crisis event, characterised by marked decrease in proliferation rate during 2 weeks, took place around passage 80. Post-crisis Hu-MI cells exhibited heterogeneous morphology, possibly corresponding to particular phenotypes that may have emerged during the crisis period. The appearance of the cell cultures shortly after the crisis event is illustrated in Figure 1a. Cultures were composed of two cell types. Epithelial-like cuboidal cells, probably identical to pre-crisis Hu-MI cells, were engulfed into islands by a predominant, newly emerged cell type with rather flattened and elongated morphology. After a few passages the cultures homogeneously contained such post-crisis cells, which are referred to as HuMI-T cells (Figure 1b). Since the exact passage number at which HuMI-T cells were selected could not be determined, we continued to use the same passage number as for the Hu-MI cells.

In contrast to Hu-MI cells which strictly grow anchorage-dependently, HuMI-T cells were found to be clonogenic (see below). In spite of the homogeneous morphology of HuMI-T cultures, we assumed that they might actually be endowed with different growth properties. Considering that clonogenicity correlates best with *in vivo* tumorigenicity, we attempted to establish possible tumorigenic sublines of HuMI-T cells by isolating individual large-sized colonies growing in soft agar at passage 105. Cells were expanded and, when heterogeneous morphology was observed, they were further selected using the limiting dilution technique. Six sublines were selected (data not shown), three of which

**Figure 1** Phase contrast photomicrographs illustrating morphology of (**a**) post-crisis Hu-MI cells at passage 89 and (**b**) HuMI-T cells at passage 107. Scale bars: 20 μm.

senesced after 4–7 passages. One of the continuous sublines, HuMI-TTu1, was found to be tumorigenic in nude mice (see below). HuMI-TTu1 cells were considered to be at passage 1 at the time of selection from soft agar cultures. At early passages, HuMI-TTu1 cultures were characterised by morphologically small round-cuboidal cells, together with a few round and rather large cells (Figure 2a). At later passages (around passage 20) these large cells had disappeared and HuMI-TTu1 cultures consisted of small, rather round-shaped cells that formed tight monolayers (Figure 2b). Apart from their epithelial-like aspect they did not morphologically resemble to either Hu-MI or HuMI-T lines. Establishment of HuMI-T and HuMI-TTu1 cells is depicted in Figure 3.

The above mentioned crisis event took place in a batch which was the only serially transferred culture of Hu-MI cells in our laboratory at that period. No comparable phenomenon was observed to occur again in samples of the same or other batches of Hu-MI cells placed into long-term culture. Chromosomal markers common to Hu-MI, HuMI-T and HuMI-TTu1 cells (see below), as well as strong and uniform expression of large T-antigen in all lines (see below) exclude this 'crisis' event to be related to culture contamination. DNA fingerprinting of Hu-MI (passage 42), HuMI-T (passage 118) and HuMI-TTu1 (passage 18 and 32) has been performed and has definitely confirmed their common origin.

*Growth properties*

The population doubling time of HuMI-T and HuMI-TTu1 cells was 46 and 37 h, respectively. At confluence HuMI-T and HuMI-TTu1 cells had a 1.8 or 2.3-fold higher saturation density than Hu-MI cells, i.e. $2.2 \times 10^5$ and $2.8 \times 10^5$ cells $cm^{-2}$, respectively.

The parental line Hu-MI does not form colonies in soft agar (Garcia *et al.*, 1991). We repeated clonogenic assays in methylcellulose and confirmed that their growth remained indeed strictly anchorage-dependent at early and late passages, in our laboratory in Epalinges (A. Yilmaz) as well as in Los Angeles (A. Imam). In contrast, HuMI-T and HuMI-TTu1 cells were found to be clonogenic. HuMI-T and HuMI-TTu1 cells had maximal cloning efficiencies (C.E.) of 13.7% and 27.3%, respectively, in methylcellulose supplemented with 10% FCS. To measure the dependence of anchorage-independent growth of HuMI-T and HuMI-TTu1 cells on the concentration of FCS, we performed methylcellulose assays using 1, 5 and 10% FCS. The C.E. of HuMI-T cells was not significantly affected by varying FCS concentrations between 5 and 10% ($P = 0.05938$) (Figure 4). When compared to C.E. in 1% FCS, colony formation increased by about 70% in 5% FCS and 100% in 10% FCS. HuMI-TTu1 cells, however, were found to be more dependent on high levels of FCS concentrations, with C.E. increasing by about 190% in 5% FCS ($P = 0.0024$) and 300% in 10% FCS ($P = 0.0022$).

**Figure 3** Summarised overview of selection and growth properties of Hu-MI, HuMI-T and HuMI-TTu1 lines.

**Figure 2** Phase contrast photomicrographs illustrating morphology of (**a**) HuMI-TTu1 cells at passage 4 and (**b**) HuMI-TTu1 cells at passage 55. Scale bars: 20 μm.

*Tumorigenicity and xenograft histology*

We tested the tumorigenic potential of HuMI-T and HuMI-TTu1 cells by injecting $10^7$ cells of each respective population

**Figure 4** C.E. of HuMI-T (open bars) and HuMI-TTu1 (filled bars) in methylcellulose. Values indicate the increase in colony-formation compared to 1% FCS. Representative for four experiments.

s.c. into nude mice. HuMI-T cells were inoculated into five mice at passage 130 and no tumour growth was observed for more than 5 months. HuMI-TTu1 cells were injected at passage 24 resulting in rapidly growing tumours in 3/4 nude mice. Tumours tended to ulcerate superficially at 42 days after inoculation and eventually reached an average volume of 0.47 $cm^3$ (s.d. = 0.082).

Histological examination of HuMI-TTu1 cells growing as s.c. xenografts revealed the presence of a particular pattern of differentiation. As illustrated in Figure 5, pleomorphic nuclei and numerous mitotic figures were seen closely associated with extensions of the murine stroma newly elicited by tumour cells (St). At distance from such proliferative regions, cells became larger and paler, with distinct pericellular polygonal irregular borders (☆: pavement appearance). Necrotic areas (N) were found to consist of an accumulation of dense material together with cellular debris and pyknotic nuclei, suggestive of keratinocyte-type of differentiation. No metastatic foci could be detected by histological examination of both regional axillary lymph node or lung sections.

Two other sublines, which had been selected from individual anchorage-independent colonies of HuMI-T cells (see above) were tested in five nude mice each. They were non-tumorigenic.

*Phenotypic characterisation*

The expression of specific epithelial antigens by HuMI-T and HuMI-TTu1 cells was determined by immunohistochemistry. Like the parental line Hu-MI (Garcia *et al.*, 1991), HuMI-T and HuMI-TTu1 cells strongly and uniformly expressed keratin 18 at all passages tested (89 to 125 for HuMI-T and 9 to 49 for HuMI-TTu1 cells). Expression of keratin 19, specifically characteristic of mammary epithelial cells of luminal origin, decreases from 100% in the earliest passage to 40% at passage 80 of HuMI cells (Garcia *et al.*, 1991). HuMI-T and HuMI-TTu1 cells were tested at early and later passages and were found to be negative for keratin 19 (data not shown).

Hu-MI, HuMI-T and HuMI-TTu1 cells have been subjected to immunostaining using antibodies to antigens of MFGM. Hu-MI (Figure 6a) and HuMI-TTu1 (Figure 6b) cells reacted strongly and uniformly with antibodies to MFGM. HuMI-T cells, however, reacted relatively weakly with this antibody. All three cell lines were EMA-positive.

Nuclei of Hu-MI, HuMI-T and HuMI-TTu1 cells were strongly and uniformly immunoreactive for large-T antigen. As for Hu-MI cells (Garcia *et al.*, 1991) there was no immunostaining of HuMI-T and HuMI-TTu1 cells using an anti-carcinoembryonic antigen antibody (data not shown).

**Figure 5** Histological section of HuMI-TTu1 cells growing as s.c. xenograft in nude mice. Scale bar: 100 μm.

**Figure 6** Immunoperoxidase staining of (**a**) Hu-MI cells at passage 40 and (**b**) HuMI-TTu1 cells at passage 20 with anti-MFGM antibody.

*Chromosome analysis*

Hu-MI cells were characterised by both structural and numerical chromosome aberrations at passage 29. The modal number was 74,X. At least two normal copies of each chromosome were found, except for a single chromosome X. Nine markers involving chromosomes 1, 3, 6, 8, 11 and 12 were always present. They can be described as follows: $M_1$: del(1q), $M_2$: del(1p), $M_3$: del(3p), $M_4$: t(3q;?), $M_5$: t(6p?;12q), $M_6$: der(8), $M_7$: t(11q;?), $M_8$: small isochromosome, $M_9$: small acrocentric.

Cytogenetic analysis of HuMI-T cells at passage 130 revealed two cell populations; 65% of the cells showed a chromosome number ranging from 59–68, X (3n−), and 35% a total number of 130, XX chromosomes (6n−). At least one normal copy of each chromosome was present, except for chromosome 13 which was missing in about 25% of the analysed cells. Twelve markers were found in at least 60% of the metaphases observed. They involved chromosomes 1, 7, 8, 11, 13, 14, 15 and are described as follows: $M_1$: del(1q), $M_2$: t(1p;7p?), $M_3$: t(1q;15), $M_4$: i(1q), $M_5$: del(7q?), $M_6$: der(8), $M_7$: i(11q), $M_8$: $13p^+$, $M_9$: $14p^+$, $M_{10}$: t(15;?), $M_{11}$: small acrocentric, $M_{12}$: ring chromosome. $M_1$, $M_6$ and $M_9$/$M_{11}$ are the only common markers of Hu-MI and HuMI-T cells.

HuMI-TTu1 cells were analysed at passages 20 (data not shown) and 45, namely before and after stabilisation of their morphology. They revealed both structural and numerical aberrations. At late passage the modal number was 66,XX with 10% of the cells exhibiting hexaploidy. No normal copies of chromosomes 1, 3 and 22 were observed. Twenty markers were identified. Chromosome 1 was involved in the formation of three markers, resulting in monosomy 1p. Chromosome 3 was involved in the formation of three more markers resulting in monosomy 3p. Other chromosomes involved in marker formation were 5, 7, 8, 10, 11 and are described as follows: $M_9$: t(1p;?), $M_2$: t(1q;?), $M_3$: i(1q), $M_4$:

der t(1p;3q), $M_5$: t(3q;10q), $M_6$: t(3p;?), $M_7$: der t(5;11), $M_8$: del(7q), $M_9$: der(8), $M_{10}$: 10p$^-$, $M_{11}$: i(11q), $M_{12}$: t(13;?), $M_{13}$: small acrocentric, $M_{14}$: small isochromosome, $M_{15}$ to $M_{20}$ could not be properly ascertained but were constantly found at both passages analysed. One half of 14 markers which could be determined at passage 20 were found also at late passage ($M_1$, $M_4$, $M_5$, $M_{11}$, $M_{12}$, $M_{15}$, $M_{16}$) and 13 new markers had appeared after morphological stabilisation of HuMI-TTu1 cells. Markers $M_9$ and $M_{14}$ were also present in the parental Hu-MI line. Markers $M_8$, $M_9$, $M_{11}$, $M_{13}$, and $M_{14}$ were commonly seen in HuMI-T cells.

### Discussion

Cells residing in the terminal ductal lobular units and belonging to the luminal epithelial lineage are the likely precursor cells of invasive breast cancer. Several mammary epithelial cell lines have been established by immortalising luminal cells found in early lactation samples, through introduction of the SV40 large T-antigen (Garcia *et al.*, 1991; Bartek *et al.*, 1990; Chang *et al.*, 1982). They retain a phenotype compatible with the putative role of breast cancer precursor cells and are neither clonogenic in soft agar nor tumorigenic in nude mice. The expression of the viral large T-antigen induces continuous production of growth signals in several cell systems (Bartek *et al.*, 1991; Lemoine *et al.*, 1989; Poirier *et al.*, 1988) via binding to a variety of proteins endowed with antiproliferative functions, such as the p53 protein (Lane & Crawford, 1979; Linzer & Levine, 1979) and the retinoblastoma gene product (Huang *et al.*, 1990; Hu *et al.*, 1990). SV40 large T-antigen is capable of transforming cells and causing tumours in the absence of any cooperating oncogene (Green, 1989; Choi *et al.*, 1983) but SV40-infected normal human epithelial cells from various tissues have repeatedly been reported to be non-tumorigenic (Cussenot *et al.*, 1991; Garcia *et al.*, 1991; Bartek *et al.*, 1990; Chang, 1986) or at best to grow anchorage-independently (Caron de Fromentel *et al.*, 1985). Although generally considered to be a rather unlikely event, malignant transformation of normal cells from mammoplasty reduction surgical samples has been observed upon SV40-immortalisation (Berthon *et al.*, 1992). Our results indicate that it may also occur in the human luminal mammary epithelial cells. The growth characteristics of HuMI-T (clonogenic but no tumorigenic growth) and HuMI-TTu1 (clonogenic as well as tumorigenic growth) lines further indicate that the initial immortalisation with SV40 DNA alone may induce random genetic changes in the host DNA resulting in differentially transformed cell populations.

Earlier attempts to obtain cell lines from milk cultures by SV40-immortalisation have resulted in the establishment of either keratin 19-negative (Caron de Fromentel *et al.*, 1985) or keratin 19-positive cell lines (Bartek *et al.*, 1990), thus suggesting that different cell populations had been immortalised. Indeed, the proportion of keratin 19-positive Hu-MI cells already decreases from 100% at early passages to 40% at passage 80 (Garcia *et al.*, 1991). Finally, both sublines HuMI-T and HuMI-TTu1 were keratin 19-negative. Luminal epithelial cells differentiate from cells located in the basal layer, keratin 19-negative luminal cells deriving from such cells are precursors to the 19-positive more differentiated luminal cells (Bartek *et al.*, 1991). In our sublines, this would mean that the Hu-MI cell line contained an undetectably small proportion of keratin 19-negative precursor cells at early passages which, later on, grew with a selective advantage in culture. On the other hand, such differentiation may of course be two-directional according to culture conditions. This is currently being investigated with our cells.

HuMI-T and HuMI-TTu1 cells were selected following a period of markedly decreased proliferation rate. This 'crisis' period, commonly seen with SV40-transformed fibroblasts, occurred in one culture only and should thus be much rarer for luminal epithelial cells even though the initial immortalising agent was the same. Indeed, in a series of SV40-transformed milk epithelial cells studied by Bartek *et al.* (1991) only 5/17 lines underwent a 'crisis' period after which their growth ceased; these five lines were comprised of elongated cells found in small numbers in milk, likely to originate from a cell type unrelated to the luminal cell. The events which have taken place in Hu-MI cells during the 'crisis' stage are not easily amenable to analysis. An accumulation of spontaneous chromosomal rearrangements affecting oncogenes or tumour suppressor genes may have resulted in a period of instability conferring a selective growth advantage to newly emerged cell types. Arbitrary and randomly destabilising effects on the mammary cell genome may well be ascribed to the initial event of immortalisation by SV40 itself. A correlation between copy number or integration site of SV40 DNA and induction of distinct growth properties cannot be ruled out. Hu-MI cells contain two copies of SV40 DNA integrated into the cellular genome and 12–14 copies of free SV40 DNA (Garcia *et al.*, 1991). Identification of the number of copies and integration sites of SV40 DNA in our sublines is under investigation.

Chromosomal alterations are important in the oncogenic process (Weinberg, 1989; Bishop, 1987). Cytogenetic analyses of primary and metastatic tumours have outlined genetic aberrations frequently involving chromosomes 1, 6, 7, 11, 13, 17, and 18 (Cropp *et al.*, 1990; Callahan & Campbell, 1989; Mackay *et al.*, 1988; Ali *et al.*, 1987; Lundberg *et al.*, 1987). The relation between a single mutation and the resulting alteration of growth properties or stage of transformation is difficult to examine. Interestingly, Hu-MI, HuMI-T and HuMI-TTu1 cells all present aberrations of chromosomes 1, 8 and 11, possibly indicative of some intermediate and necessary, but not sufficient steps between normality and malignancy in our model. Whereas a deletion of chromosome 7 was common in HuMI-T and HuMI-TTu1 cells, aberrations involving chromosomes 5, 10 and an absence of a normal chromosome 22 were unique to the tumorigenic cell line HuMI-TTu1.

No established *in vitro* cell system allows at present understanding all the complexity of mammary tumorigenesis. Most commonly used breast cancer lines are derived from cells from pleural effusions; thus, they result from selective processes and are not necessarily characteristic of cells in primary tumours or solid metastases (Dickson & Lippman, 1987). Lines from normal breast tissue, on the other hand, are established through immortalisation processes or originate from fibrocystic lesions (Soule *et al.*, 1990; Paine *et al.*, 1992) and are not truly representative of normal breast cell behaviour. Though bearing the technically required disadvantage of being immortalised, Hu-MI, HuMI-T and HuMI-TTu1 lines, due to their common origin and the different stages of malignant transformation of breast cancer precursor cells they represent, may well provide a novel *in vitro* system for future comparative studies on growth regulation of breast cells in the process of malignant progression.

This work was supported in part by a grant from Foundation Muschamp to A.Y. and the Swiss National Science Foundation.

We thank Dr I. Garcia who kindly provided the Hu-MI cell line. The help of Dr W. Bär and Dr A. Kratzer, Institute of Forensic Medicine, Zürich, who performed DNA fingerprinting, is gratefully acknowledged. We also thank M. Baumgartner, J. Bamat, L. Bucher, M. Allegrini and P. Dubied for excellent technical assistance.

### References

ALI, I.U., LIDEREAU, R., THEILLET, C. & CALLAHAN, R. (1987). Reduction to homozygosity of genes in chromosome 11 in human breast neoplasia. *Proc. Natl Acad. Sci. USA*, **84**, 185-188.

BAND, V., ZAJCHOWSKI, D., KULESA, V. & SAGER, R. (1990). Human papilloma virus DNAs immortalize normal human mammary epithelial cells and reduce their growth-factor requirements. *Proc. Natl Acad. Sci. USA*, **87**, 463-467.

BARTEK, J., BARTKOVA, J., KYPRIANOU, N., LALANI, E.-N., STASKOVA, Z., SHEARER, M., CHANG, S. & TAYLOR-PAPADIMITRIOU, J.-T. (1991). Efficient immortalization of luminal epithelial cells from human mammary gland by introduction of simian virus large tumor antigen with recombinant retrovirus. *Proc. Natl Acad. Sci. USA*, **88**, 3520-3524.

BARTEK, J., BARTKOVA, J., LALANI, E., BREZINA, V. & TAYLOR-PAPADIMITRIOU, J. (1990). Selective immortalization of a phenotypically distinct epithelial cell type by microinjection of SV40 DNA into cultured human milk cells. *Int. J. Cancer*, **45**, 1105-1112.

BARTEK, J., DURBAN, E.M., HALLOWES, R.C. & TAYLOR-PAPADIMITRIOU, J. (1985*a*). A subclass of luminal epithelial cells in the human mammary gland, defined by antibodies to cytokines. *J. Cell Sci.*, **75**, 17-33.

BARTEK, J., TAYLOR-PAPADIMITRIOU, J., MILLER, N. & MILLIS, R. (1985*b*). Patterns of expression of keratin 19 as detected with monoclonal antibodies in human breast tissues and tumours. *Int. J. Cancer*, **36**, 299-306.

BERTHON, P., GOUBIN, G., DUTRILLAUX, B., DEGEORGES, A., FAILLE, A., GESPACH, C. & CALVO, F. (1992). Single step transformation of human breast epithelial cells by SV40 large T oncogene. *Int. J. Cancer*, **52**, 92-97.

BISHOP, J.M. (1987). The molecular genetics of cancer. *Science*, **235**, 305-311.

BRIAND, P., PETERSEN, O.W. & VAN DEURS, B. (1987). A new diploid non-tumorigenic human breast epithelial cell line isolated and propagated in chemically defined medium. *In Vitro*, **23**, 181-188.

BUEHRING, G.C. (1972). Culture of human mammary epithelial cells: keeping abreast with a new method. *J. Natl Cancer Inst.*, **49**, 1433-1434.

CALLAHAN, R. & CAMPBELL, G. (1989). Mutations in human breast cancer: an overview. *J. Natl Cancer Inst.*, **81**, 1780-1786.

CARON DE FROMENTEL, C., NARDEUX, P.C., SOUSSI, T., LAVIALLE, C., ESTRADE, S., CARLONI, G., CHANDRASEKARAN, K. & CASSINGENA, R. (1985). Epithelial HBL-100 cell line derived from milk of an apparently healthy woman harbours SV40 genetic information. *Exp. Cell Res.*, **160**, 83-94.

CHANG, S.E. (1986). *In vitro* transformation of human epithelial cells. *Biochim. Biophys. Acta*, **823**, 161-194.

CHANG, S.E., KEEN, J., LANE, E.B. & TAYLOR-PAPADIMITRIOU, J. (1982). Establishment and characterization of SV40-transformed human breast epithelial cell lines. *Cancer Res.*, **42**, 2040-2053.

CHOI, K.H., TEVETHIA, S.S. & SHIN, A. (1983). Tumor formation by SV40-transformed human cells in nude mice: the role of SV40 T-antigens. *Cell Genet.*, **36**, 633-640.

CROPP, C.S., LIDERAU, R., CAMPBELL, G., CHAMPENE, M.H. & CALLAHAN, R. (1990). Loss of heterozygosity on chromosome 17 and 18 in breast carcinoma: two additional regions identified. *Proc. Natl Acad. Sci. USA*, **87**, 7737-7741.

CUSSENOT, O., BERTHON, P., FAILLE, A., BERGER, R., MOWSZOWICS, I., TEILLAC, P., LEDUC, A. & CALVO, F. (1991). Immortalization of human adult normal prostatic epithelial cells by liposomes containing SV40. *J. Urol.*, **143**, 881-886.

DICKSON, R.B. & LIPPMAN, M.E. (1987). Estrogenic regulation of growth and polypeptide growth factor secretion in human breast cancer. *Endocrine Rev.*, **8**, 29-43.

ELIASON, J.F., AAPRO, M.S., DECREY, D. & BRINK-PETERSEN, M. (1985). Non-linearity of colony formation by human tumor cells from biopsy samples. *Br. J. Cancer*, **52**, 311-318.

GARCIA, I., BRANDT, D., WEINTRAUB, J., ZHOU, W. & AAPRO, M. (1991). Loss of heterozygosity for the short arm of chromosome 11 (11p15) in human milk epithelial cells immortalized by microinjection of SV40 DNA. *Cancer Res.*, **51**, 294-300.

GREEN, M.R. (1989). When the products of oncogenes and antioncogenes meet. *Cell*, **56**, 1-3.

HU, Q., DYSON, N. & HARLOW, E. (1990). The regions of the retinoblastoma protein needed for binding to adenovirus E1A or SV40 large T. *EMBO J.*, **9**, 1147-1155.

HUANG, S., WANG, N.P., TSENG, B.Y., LEE, W.H. & LEE, E.H.P. (1990). Two distinct and frequency mutated regions of retinoblastoma protein are required for binding to SV40 T antigen. *EMBO J.*, **9**, 1815-1822.

IMAM, A. & TÖKÉS, Z.A. (1981). Immunoperoxidase localization of glycoprotein on plasma membrane of secretory epithelium from human breast. *J. Histochem. Cytochem.*, **29**, 581-584.

IMAM, A., LAURENCE, J.R. & NEVILLE, A.M. (1981). Isolation and characterization of a major glycoprotein from milk-fat-globule membrane of human breast milk. *Biochem. J.*, **193**, 47-53.

IMAM, A., TAYLOR, C.R. & TÖKÉS, Z.A. (1984). Immunohistological study of the expression of human milk-fat-globule membrane glycoprotein 70. *Cancer Res.*, **44**, 2016-2022.

LANE, D.P. & CRAWFORD, L.V. (1979). T antigen is bound to a host protein in SV40-transformed cells. *Nature*, **278**, 261-263.

LEMOINE, N.R., MAYALL, E.S., JONES, T., SHEER, D., MCDERMID, S., KENDALL-TAYLOR, P. & WYNFORD-THOMAS, D. (1989). Characterisation of human thyroid epithelial cells immortalized *in vitro* by simian virus SV40 DNA transfection. *Br. J. Cancer*, **60**, 897-903.

LINZER, D.I.H. & LEVINE, A.J. (1979). Characterization of a 54 K dalton cellular SV40 tumor antigen present in SV40-transformed and uninfected embryonal carcinoma cells. *Cell*, **17**, 43-52.

LUNDBERG, C., SKOOG, L., CAVENEE, W.K. & NORDENSKJÖLD, M. (1987). Loss of heterozygosity in human ductal breast tumors indicates a recessive mutation on chromosome 13. *Proc. Natl Acad. Sci. USA*, **84**, 2372-2376.

MACKAY, J., STEEL, C.M., ELDER, P.A., FORREST, A.P.M. & EVANS, H.J. (1988). Allele loss on short arm of chromosomes 17 in breast cancer. *Lancet*, **2**, 1384-1385.

O'HARE, M.J. (1991). Breast cancer. In *Human Cancer in Primary Culture*, Masters, J.R.W. (ed.). pp. 271-286. Kluwer Academic Publishers: Netherlands.

PAINE, T.M., SOULE, H.D., PAULEY, R.J. & DAWSON, P.J. (1992). Characterization of epithelial phenotypes in mortal and immortal human breast cells. *Int. J. Cancer*, **50**, 463-473.

PATHAK, S. (1976). Chromosome banding techniques. *J. Reprod. Med.*, **17**, 25-28.

POIRIER, V., TYLER, S.J., BROWN, K.W., SHAW, A.P.W. & MAITLAND, N.J. (1988). SV40 transfection of human epithelial cells and stability of chromosome 11. *Int. J. Cancer*, **42**, 887-894.

RUSSO, J., CALAF, G., ROI, L. & RUSSO, I.H. (1987). Influence of age and gland topography on cell kinetics of normal human breast tissue. *J. Natl Cancer Inst.*, **78**, 413-418.

SOULE, H.P., MALONEY, T., WOLMAN, S., PETERSON, W., BRENTZ, R., MCGRATH, C.M., RUSSO, J., PAULEY, R., JONES, R. & BROOKS, S.C. (1990). Isolation and characterization of a spontaneously immortalized human breast epithelial cell line, MCF-10. *Cancer Res.*, **50**, 6087-6094.

STAMPFER, M.R. & BARTLEY, J.C. (1985). Induction of transformation and continuous cell lines from normal human mammary epithelial cells after exposure to benzo(*a*)pyrene. *Proc. Natl Acad. Sci. USA*, **82**, 2394-2398.

TAYLOR, C.R. (1978). Immunoperoxidase techniques. *Arch. Pathol. Lab. Med.*, **102**, 113-121.

TAYLOR-PAPADIMITRIOU, J. & LANE, E.B. (1987). Keratin expression in the mammary gland. In Neville, M.C. & Daniel, C.W. (ed.). *The Mammary Gland*, Plenum Press: New York, pp. 181.

TAYLOR-PAPADIMITRIOU, J., SHEARER, M. & TILLY, R. (1977). Some properties of cells cultured from early-lactation human milk. *J. Natl Cancer Inst.*, **58**, 1563-1571.

TAYLOR-PAPADIMITRIOU, J., STAMPFER, M., BARTEK, J., LEWIS, A., BOSHELL, M., LANE, E.B. & LEIGH, I.M. (1989). Keratin expression in human mammary epithelial cells cultured from normal and malignant tissue: relation to *in vivo* phenotype and influence of medium. *J. Cell Sci.*, **94**, 403-413.

WEINBERG, R.A. (1989). Oncogenes, antioncogenes, and the molecular bases of multistep carcinogenesis. *Cancer Res.*, **49**, 3713-3721.

WELLINGS, S.R., JENSEN, H.M. & MARCUM, R.G. (1975). An atlas of subgross pathology of the human breast with special reference to possible precancerous lesions. *J. Natl Cancer Inst.*, **55**, 231-273.